# Solid Cancers and Rheumatoid Arthritis

**DOI:** 10.3390/cancers15225441

**Published:** 2023-11-16

**Authors:** George D. Kalliolias, Efthimia K. Basdra, Athanasios G. Papavassiliou

**Affiliations:** 1 Hospital for Special Surgery, Arthritis & Tissue Degeneration, New York, NY 10021, USA; kallioliasg@hss.edu; 2 Department of Medicine, Weill Cornell Medical College, New York, NY 10065, USA; 3 Regeneron Pharmaceuticals, Inc., Tarrytown, NY 10591, USA; 4 Department of Biological Chemistry, Medical School, National and Kapodistrian University of Athens, 11527 Athens, Greece; ebasdra@med.uoa.gr

## 1. Introduction

Since the initial observation that patients with rheumatoid arthritis (RA) have an excess risk of developing hematologic malignancies [1], accumulating evidence has established a similar concept for solid tumors [2]. The all-solid tumors risk is ~20% higher in RA patients compared to the general population, with men displaying 1.24 times higher incidence than women [3]. Notably, there are site-specific differences in solid cancer risks, with an increased incidence of solid tumors associated with smoking or oncogenic viral infections (i.e., lung cancer, urinary bladder, kidney, upper urinary tract, ear–nose–throat (ENT) cancers, and cervical cancer) and decreased incidence for breast and colon cancers [4]. The key mechanisms that may explain the differential risk for solid tumors in RA are listed in Table 1.

Over the last years, the approval of new classes of medications has revolutionized the therapeutic paradigm for RA [5]. The current treatment armamentarium includes conventional synthetic disease-modifying antirheumatic drugs (cs-DMARDs), biologic DMARDs (b-DMARDs), and targeted synthetic DMARDs (ts-DMARDs) such as the class of Janus kinase inhibitors (JAKi) [6]. The new standard of care for RA achieves robust immunomodulation with a profound benefit on disease burden, but at the same time raises concerns about its impact on the immunosurveillance against tumors and oncogenic viruses [4]. The continuously changing practices in RA management (i.e., new classes of DMARDs and the diminished need for nonsteroid anti-inflammatory drugs (NSAIDs)), together with life-style modifications (e.g., changes in smoking habits, Westernized diet), and new preventive medicine practices (for example, broad vaccination for human papillomavirus (HPV)) may impact the risks for solid tumor development in RA patients. Cancer development is a low-frequency event which needs a long time to develop. In this context, there is a continuous need for the re-evaluation of solid cancer risk in RA, with new studies including larger numbers of RA patients exposed to the new classes of DMARDs with longer periods of follow-up. Here, we summarize the recent developments in the field of solid tumor surveillance in RA.

## 2. Increased Risk for Lung Cancer

Three recently published studies, from Sweden [7], South Korea [8], and France [3], including very large numbers of RA patients (*n* > 44,000, *n* > 50,000, and *n* > 275,000, respectively) exposed to all the available classes of DMARDs and with long follow-up periods (median follow-up = 7.3, 4.5, and 8.7 years, respectively), have confirmed the observation made from older studies [2] that patients with RA display 50–100% increased risk for lung cancer compared to the general population. This finding has been explained on the basis of shared etiology between RA and lung cancer [4]. Smoking, chronic lung inflammation, and interstitial lung disease (ILD) have been the proposed etiologic links among the two diseases [4,9].

Smoking increases >20-fold the risk of developing RA in genetically predisposed individuals (carriers of shared epitope or risk variants of *PTPN22*) [10]. According to the *mucosal origin hypothesis*, the initiating events of RA pathogenesis resulting in the break of self-tolerance and the production of anticitrullinated peptide antibodies (ACPAs) may occur in the lung and be triggered by smoking [11]. Cigarette smoking induces local lung inflammation and the activation of the enzyme peptidyl-arginine deiminase (PADI), resulting in the de novo citrullination of lung proteins and emergence of citrullinated neoepitopes [10,11]. Among RA patients, the relative risk for the development of lung cancer is 19.24 for current and 11 for past smokers compared to RA patients who have never smoked [12].

The role of sex, seropositivity, disease duration, and ILD as risk factors for lung cancer development in RA patients has been investigated in several studies. The risk for lung cancer in RA is higher in males compared to females [3], a finding that has been explained primarily because of the higher exposures of males to smoking. The Swedish study has also identified seropositivity for ACPAs and/or rheumatoid factor (RF) as an independent risk factor for lung cancer development, that even when adjusted for smoking, increases 2–6 times the incidence of lung cancer [7]. This new observation has not been confirmed by the South Korean study [8], but this may suggest that regular CT lung screening should be considered for RA patients who are seropositive and have ever been smokers. Regarding the impact of RA disease duration, according to the Swedish study, during the first 5 and 20 years after the diagnosis of RA, 1% and 3%, respectively, of RA patients were diagnosed with lung cancer [7]. Notably, another study has shown that the risk of lung cancer was lower among patients with an RA disease duration of ≥10 years compared to <1 year [12]. Additionally, ILD is associated with a higher risk of lung cancer, and clinically significant ILD occurs in 5–10% of patients with RA [9,13]. Data from the ORAL Surveillance trial suggest that RA patients with a history of chronic lung disease (ILD or chronic obstructive pulmonary disease) display 2.63-times higher risk for lung cancer compared to RA patients with no history of chronic lung disease [12,14].

The impact of background treatments on the risk for lung cancer development in RA patients has been evaluated recently. The French study [3] compared RA patients exposed to cs-DMARDs (*n* > 78,000), tumor necrosis factor inhibitors (TNFi) (*n* > 65,000), abatacept (*n* > 14,000), rituximab (*n* > 13,000), and anti-IL6 receptor (anti-IL6R) (*n* > 14,000), showing that the highest incidence of lung cancer was observed in RA patients exposed to abatacept (Standardized Incidence Ratio (SIR) = 2.10) and rituximab (SIR = 1.68), followed by RA patients exposed to cs-DMARDs (SIR = 1.42), TNFi (SIR = 1.41), and anti-IL6R (SIR = 1.15). JAKi is the most recently approved class of DMARDs [5], and data on their impact on lung cancer risk are more limited due to lower numbers of exposed RA patients and shorter follow-up [12,14,15,16]. The ORAL Surveillance Trial has shown a higher incidence of lung cancer in RA patients exposed to tofacitinib (1.08%, 30 cases of lung cancer among 2911 RA patients) than in RA patients exposed to TNFi (0.48%, 7 cases of lung cancer among 1451 RA patients) [12]. On the other hand, it was recently reported that RA patients exposed to tofacitinib displayed a lower incidence of ILD compared to RA patients exposed to adalimumab, abatacept, rituximab, or tocilizumab [13]. In this context, more studies are required to better evaluate the potential long-term impact of JAKi on the risk of lung cancer development.

## 3. Increased Risks for Other Site-Specific Solid Tumors

Over the last 30 years, more than 18 studies have provided conflicting data on the risk for cervical cancer. In 2015, a meta-analysis of 15 studies has estimated a modestly decreased risk (total pooled SIR = 0.87) [2]. More recent studies with larger numbers of RA patients and longer follow-up periods have shown consistently about 1.5–1.8-times higher risk for cervical intraepithelial neoplasia (CIN) 1–3 and cervical cancer [17,18]. A nationwide study from Sweden has shown that women exposed to TNFi had about two-times higher risk for invasive cervical carcer compared to biologics-naïve women with RA [18]. Cervical dysplasia and invasive cervical cancer arise from infection with HPV [19], and the immunosuppressive DMARDs used to treat RA may impair the clearance of the oncogenic HPV [4]. Shared etiology (i.e., smoking) [20] could be an additional explanation for the association of RA with cervical dysplasia and cancer.

Cumulative evidence over the years suggests that RA patients also display increased risk for skin cancers, including melanoma and nonmelanoma skin cancers (NMSCs) [2,3,21,22,23,24,25]. Therefore, periodic dermatological screening is recommended in RA patients for the early detection of skin cancers. Regarding melanoma, a meta-analysis pooling 21 studies with conflicting results has shown a modestly increased risk (total pooled SIR = 1.23) for RA patients [2]. A recent very large study (*n* > 257,000 RA patients) has also shown an increased risk (SIR = 1.37) for melanoma in RA patients in the French population [3]. Concerns about the impact of immunosuppressive DMARDs on immunosurveillance against the immunogenic melanoma have been explored in a collaborative project of 11 European biologic registries collecting data from >130,000 RA patients [21]. The results of this study have shown that the incidence of melanoma in RA patients who were biologic-naïve, TNFi-exposed, rituximab-exposed, tocilizumab-exposed, and abatacept-exposed was slightly higher in all RA treatment groups compared to the general population [21]. Also, the comparison between TNFi-exposed versus biologic-naïve RA patients has shown a slightly but not significantly increased incidence rate ratio (IRR = 1.14) of invasive melanoma in TNFi-exposed patients [21], alleviating the concerns raised from prior smaller studies [22,23].

Concerning prostate cancer, the second most commonly occurring cancer in men, a meta-analysis pooling 17 studies over a 20-year period (1993 to 2013) has shown a slightly increased risk (total pooled SIR = 1.15) in RA patients [2]. Along the same lines, a recently published French study has shown insignificant differences in the incidence of prostate cancer between RA patients and the general population (SIR = 1.08) [3].

## 4. Decreased Risks for Breast and Colon Cancers

Notably, the incidence of colon and breast cancers has been consistently reported to be lower in RA patients compared to the general population [2,3]. For colon cancer, the lower risk has been attributed to the impact of nonsteroid anti-inflammatory drugs (NSAIDs) that have a proven effect on reducing the risk for developing colon cancer [26] and are commonly utilized by RA patients for arthritic pain control. Regarding the lower risk for breast cancer in RA, according to a recent study that adjusted the model of risk estimations for several breast cancer risk determinants, the explanation remains unknown and cannot be attributed to any of the traditional breast cancer risk factors [27]. Despite the decreased risk of breast cancer in RA, it is worth mentioning the potential impact of glucocorticoids (GCs) on the risk for breast cancer. GCs represent a substantial part of the therapeutic armamentarium for RA treatment and are widely administered as a bridging therapy in combination with cs-DMARDs. Nevertheless, given that glucocorticoid receptor (GR) signaling could theoretically augment breast cancer risk and progression by triggering insulin resistance and facilitating immunosuppression [28,29], and that GR polymorphisms might affect GCs action, thus interfering with the pathogenetic mechanism of RA [30], the future position GCs may have in patients with RA should be re-evaluated in order to ameliorate the balance between efficacy and long-term safety.

## Figures and Tables

**Table 1:** Three mechanisms explaining the differential site-specific risks for cancer in RA: (1) RA and cancer share etiologic factors (e.g., smoking), (2) RA promotes site-specific carcinogenesis (e.g., RA targets the lung and induces chronic lung inflammation and interstitial lung disease), and (3) DMARDs, NSAIDs, and GCs used for the treatment of RA impact the risk for the development of site-specific cancers (e.g., DMARDs decrease immunosurveillance against tumors and oncogenic viruses permitting unopposed tumor development). RA, rheumatoid arthritis; DMARDs, disease-modifying antirheumatic drugs; NSAIDs, nonsteroid anti-inflammatory drugs; GGs, glucocorticoids; ILD, interstitial lung disease; HPV, human papillomavirus; ENT, ear–nose–throat; UIT, upper urinary tract; NMSC, nonmelanoma skin cancer.

Mechanisms			Cancer Risk
Shared Etiology Between RA and Cancer	Common exposures	Smoking	Increased risk for smoking-related cancers: Lung ENT Kidney UIT Bladder Cervical
RA Promotes Cancer	Hyperactivation of lymphoid organs		Increased risk for: Hematologic malignancies
	Chronic inflammation Tissue damage	ILD Seropositivity	Increased incidence: Lung
RA Treatment Impacts the Risk for Cancer	Immunomodulatory DMARDs	Impaired immunosurveillance against: Tumors Oncogenic Viruses (e.g., HPV)	Increased risk for: Melanoma NMSC Cervical cancer ENT cancer
	NSAIDs		Decreased risk for: Colon cancer
	GCs		Impact on the risk for: Breast cancer

## Data Availability

Data are contained within the article.
